# Gender and Body-Fat Status as Predictors of Parental Feeding Styles and Children’s Nutritional Knowledge, Eating Habits and Behaviours

**DOI:** 10.3390/ijerph15050852

**Published:** 2018-04-25

**Authors:** Małgorzata Lipowska, Mariusz Lipowski, Paweł Jurek, Anna M. Jankowska, Paulina Pawlicka

**Affiliations:** 1 Institute of Psychology, University of Gdansk, Bażyńskiego 4, 80-309 Gdansk, Poland; psypj@ug.edu.pl (P.J.); a.jankowska@ug.edu.pl (A.M.J.); paulina.pawlicka@ug.edu.pl (P.P.); 2 Department of Health Psychology, Gdansk University of Physical Education and Sport, Górskiego 1, 80-336 Gdansk, Poland; mariusz.lipowski@awfis.gda.pl

**Keywords:** overfat, obesity, nutritional knowledge, eating patterns, feeding styles

## Abstract

The home food environment is critically important for the development of children’s health-related practices. By managing dietary restrictions, providing nutritional knowledge and demonstrating eating behaviours, parents contribute to children’s food preferences and eating patterns. The present study examined nutritional knowledge, eating habits and appetite traits among 387 Polish five-year-old healthy and overfat boys and girls in the context of parental feeding styles and body-fat status. We observed that girls presented healthier eating habits than boys; however, overfat boys had better nutritional knowledge. Children’s body-fat percentage (%BF) was found to be linked with eating behaviours such as low satiety responsiveness and increased food responsiveness in girls as well as low emotional undereating and increased emotional overeating in boys. Our results revealed that overfat mothers, who were more prone to use the encouragement feeding style, rarely had daughters with increased %BF. Parents of overfat girls, however, were less likely to apply encouragement and instrumental feeding styles. Contrary to popular belief and previous studies, overfat women do not necessarily transmit unhealthy eating patterns to their children. Parents’ greater emphasis on managing the weight and eating habits of daughters (rather than sons) probably results from their awareness of standards of female physical attractiveness.

## 1. Introduction

Children’s eating patterns and preferences are an outcome of the interaction of innate and learned factors embedded in the context of parent–child interactions. Biological predispositions (e.g., innate preferences, appetite regulation mechanisms) are modified through interactions with external factors often controlled by parents (e.g., diet composition, parental feeding style). Thus, parents are important agents of the development of a child’s food preferences and intake patterns [1,2].

Parents’ efforts to promote the development of a child’s nutritional preferences and habits can be divided into three categories: when, what and how [3]: (a) when pertains to the specific timing of introducing a variety of new nutrients and ingredients [4,5], (b) what applies to diet composition and exposure to a variety of food tastes and textures [6,7], and (c) how concerns the approach to feeding in the context of parent–child interactions and the development of eating self-regulation [8,9,10]. How parents shape their children’s eating habits depends on numerous factors which operate simultaneously. First of all, parents are the most important models for health practices. Children observe and internalize parental dietary habits and other aspects of the home food environment [11,12]. If parents rarely introduce their children to meals they dislike themselves, children, consequently, dislike them too [13]. Furthermore, regular and frequent family dinners are positively associated with children’s preference for fruits and vegetables, and avoidance of processed and fried food, sugar-rich drinks, and saturated and trans fat [14].

Secondly, parents have direct control over a child’s diet by managing the quantity and quality of their food intake (e.g., introducing restrictions and making certain foods available) and through the use of certain feeding practices [15,16] which stem from parental feeding styles [17,18]. Feeding style is a parents’ attitude towards the nutrition which translates itself into a parent–child interaction during mealtime and, thus, impacts the development of a child’s eating behaviors and habits [19]. As demonstrated in much research, parental feeding styles can determine the amount of fruits, vegetables [20,21], sugar [22] and dairy [23] consumed by children and, therefore, are associated with children’s weight [24].

The relationship between parental feeding practices and children’s eating patterns described in the literature largely pertains to the practices and attitudes of mothers, often focusing less on fathers. Mothers usually have a greater impact on the development of children’s eating behaviour as they tend to be more involved in food-related decisions and the preparation of meals. For instance, unhealthy food preferences in mothers (e.g., consuming fast food, sweets and higher-energy fluids), as well as the availability of these un-nutritional foods at home, were found to be responsible for children’s preferences when it came to consumption of these types of foods [25]. As might be expected, a significant body of research indicates that maternal obesity is a substantial predictor of childhood obesity [26,27]. Nonetheless, it is crucial to acknowledge that fathers’ attitudes and practices may also contribute to a child’s health and the development of eating habits, although mothers and fathers may differ in their strategies [28]. Approaches to feeding used by mothers and fathers vary depending on a variety of factors such as their own weight-related experiences, perception of their child’s BMI [29], or the parent’s beliefs about gender differences regarding dieting and physical attractiveness [30,31,32,33].

Therefore, in order to identify gender-specific parent–child dietary intake patterns, the aim of the present study was to examine and compare nutritional knowledge, eating habits and appetite traits among five-year-old Polish boys and girls with and without excessive body-fat status in the context of gender differences in parental feeding styles and body-fat status.

The following hypotheses were tested:

**Hypothesis** **1.** *The feeding styles preferred by parents determine children’s eating habits and shape their nutritional knowledge, consequently contributing to their body-fat status;*

**Hypothesis** **2.** *Given the gender and body-fat status, of both the parents and children, distinct patterns of relationships are present between parental feeding styles and the eating habits and nutritional knowledge of girls and boys.*

Analysis of the food-related patterns of interaction within parent–child dyads will provide a better understanding of gender-related trans-generational transmission of food-related behaviors.

## 2. Materials and Methods

### 2.1. Participants

A total of *n* = 387 five-year-old children (of whom 174 were girls) and both parents of each child participated in the study. These are the participants who completed all the required tests and questionnaires. The average age of the mothers was *M* = 33.23 (standard deviation (SD) = 4.98), and the fathers *M* = 38.13 (SD = 5.93). Children were purposely selected from the same age cohort (*M_age_* = 5.6, SD = 0.21) in order to exclude the influence of age on the factors assessed in the study. The children’s eating habits, knowledge or parental feeding styles may differ depending on the child’s age. Furthermore, the age of five is an interesting time period for examining the process of trans-generational transmission of health-related practices, standards of physical attractiveness and gender stereotypes. Additionally, in order to understand the potential importance of socioeconomic status (SES) and familial factors for parental feeding practices and child food-related experiences, several other variables were controlled for, such as (a) parents’ education levels (14% had vocational education, 38% secondary education, and 48% higher education; fathers had 32%, 37%, and 31% respectively); (b) the area of residence of the participants (28% lived in villages, 16% in small towns of up to 20,000 inhabitants, 29% in towns with 20,000–100,000 inhabitants, and 26% in big cities with over 100,000 inhabitants); and (c) whether the child had siblings (36% were only-children).

### 2.2. Procedure

Subject sampling was purposive: we randomly selected 20% of the nursery schools and primary schools that run compulsory pre-school education units (the so-called “zero grade”), according to the requirements of the Ministry of National Education, from the Pomerania region of Poland (keeping the proportions city/village), and invited them to participate in the study. A positive response was obtained from 58 school directors. Prior to the study, written informed consent was obtained from 874 families who were also informed that they could discontinue their participation at any time without repercussions. Finally, only the families in which the children performed the tests and both parents completed all questionnaires were qualified for the analysis with the body composition analyzer (*n* = 387). Families who failed to complete all requested tests and questionnaires were excluded from the research and statistical analysis. Each triad was examined by the same trained researcher (a psychologist, a PhD-student, or a graduate student involved in the project). Children were tested individually at nursery/primary schools, during times in the morning set by the teacher. Children were assessed after breakfast to make sure that feelings of hunger would not possibly influence a child’s answers regarding their eating habits and knowledge about the nutritional value of food. Parents completed self-rated questionnaires at home within two weeks, and measurements of body fat status only took place at educational centers. The protocol of this study was approved by the Ethics Board for Research Projects at the Institute of Psychology, University of Gdansk, Poland (decision no. 17/2013).

We collected several different kinds of information using Dietary Knowledge and Habits (DKH), the Children’s Eating Behavior Questionnaire (CEBQ), and the Parent Feeding Style Questionnaire (PFSQ). The objective body parameters of all participants were controlled for using a body composition analyzer.

#### 2.2.1. Dietary Knowledge and Habits (DKH)

This original instrument comprises 60 illustrations of various food products classified by competent referees (dietitians) as healthy, unhealthy, or neutral to health [34]. It is used to assess the nutritional knowledge and frequency of consumption of the illustrated food (eating habits) among children aged 5 to 9. The assessment consists of two parts. In the first stage, which assesses the child’s nutritional knowledge, the researcher shows various pictures of food while the child decides to which group (healthy, unhealthy, neutral) these meals belong. The answers are classified as correct or incorrect. No earlier than two weeks after this, in the second stage of the study when the child’s eating habits are assessed, the child is shown the same pictures and asked how often he/she eats these meals (very often, only sometimes, very rarely, or never). Using the same pictures twice allows an estimate to be made of the cohesion of the child’s nutritional knowledge and their eating habits. Furthermore, this picture-based game includes elements of the thematic interview carried out with the child. The Cronbach’s reliability coefficient for DKH reached 0.79.

#### 2.2.2. Children’s Eating Behavior Questionnaire (CEBQ)

A parent-completed measure was designed to assess variation in eating behaviours and appetite traits among children. The CEBQ [35] consists of 35 items divided into food-approach behaviours (emotional overeating, enjoyment of food, food responsiveness, and desire to drink, e.g., “My child eats more when annoyed”, “My child looks forward to mealtimes”, “Even if my child is full up s/he finds room to eat his/her favourite food”, and “My child is always asking for a drink”, respectively) and food-avoidance behaviours (emotional undereating, satiety responsiveness, slowness in eating, and fussiness, e.g., “My child eats less when upset”, “My child gets full up easily”, “My child takes more than 30 min to finish a meal”, and “My child refuses new foods at first”, respectively). Each item is rated on a 5-point Likert scale that ranges from “never” to “always”. Scores were obtained by calculating the means of the items comprising each scale. Higher scores indicate a child’s greater tendency towards certain food-approach or food-avoidance behaviors. The questionnaire is a widely used measure of children’s eating behaviours as it demonstrates good internal consistency, test-retest reliability, and validity relative to other behavioural measures [35,36].

#### 2.2.3. Parental Feeding Style Questionnaire (PFSQ)

This 27-item questionnaire [37] assesses the four most common aspects of feeding styles presented by mothers and fathers: instrumental feeding (feeding children in response to their behaviour, using food as a reward, e.g., “I reward my child with something to eat when she is well-behaved”), emotional feeding (feeding children in response to their emotions, offering food to soothe the child’s negative emotions, e.g., “I give my child something to eat to make him feel better when he is upset”), prompting and encouragement to eat (encouraging children to consume a variety of foods, e.g., “I praise my child if she eats what I give her”), and control over eating (controlling the child’s food intake, determining the types and quantities of foods that children should consume, e.g., “I decide how many snacks my child should have”). The respondents are asked to choose an answer to each item from a 5-point Likert scale (from “I never do” to “I always do”). Average scores on each scale were calculated, where higher scores indicate a greater tendency for parents to use a particular feeding style. Internal reliability, test–retest reliability, and validity of the questionnaire were confirmed to be satisfactory [37]. In this study, we collected data on feeding style from both parents. We used a measurement model that assumed the compatibility of mothers’ and fathers’ tendencies in each of the four defined styles. At the same time, we assumed a correlation between the indicators of the instrumental and emotional styles.

#### 2.2.4. Body Composition Analyzer

The body-fat status of children and parents was determined using a Segmental Body Composition Monitor–Tanita BC-601. For adults, the analyser allows the measurement of indices of obesity level adjusted for muscle mass content, fat percentage (%BF), recommended daily energetic intake, basal metabolic rate, metabolic age, bone mass, and visceral fat content, but for children the instrument only measures fat percentage—thus, we have chosen %BF as the indicator of body-fat distribution [38]. Both age and sex were taken into account when determining body-fat status (i.e., overfat, healthy or underfat) [39]. As children were at the same age, the overfat indicator was a %BF of 22% for girls and 19% for boys. Among parents, %BF was calculated individually as they differed in age [40].

### 2.3. Statistical Analysis

To verify that children’s body fat is related in some way to their parents’ body fat, we compared the numbers of healthy and overfat children (separately for girls and boys) in the healthy and overfat parents groups (separately for mothers and fathers). A Pearson chi-square test was carried out to show significant differences in distribution. In order to investigate the significance of differences in the four feeding styles in the groups of healthy and overfat mothers and fathers, a series of independent sample *t* tests (for healthy and overfat parents, separately for mothers and fathers) and paired sample *t* tests (for comparisons between mothers and fathers) were carried out.

In order to test the significance of differences between healthy and overfat boys and girls, in average nutrition knowledge as well as eating habits, we performed 2 (sex) × 2 (body fat status) ANOVA (analysis of variance) with an interaction effect. To check what specific pairs of group means show differences, the Bonferroni post-hoc multiple comparison procedure was performed.

In order to verify the measurement model of parents’ feeding style that assumed the compatibility of mothers’ and fathers’ tendencies in each of the four defined styles, we used structural equation modeling (SEM). The calculations were done in R using the lavaan package [41]. To assess the goodness of fit of the model, the following criteria were used: Root Mean Square Error of Approximation (RMSEA) and Standardized Root Mean Square Residual (SRMR) smaller than 0.08 and Comparative Fit Index (CFI) larger than 0.90. For comparing means in each group (overfat boys and girls) in each of the four defined parents’ feeding styles, we used multivariate analysis of variance (MANOVA). Subsequently, the Bonferroni post-hoc multiple comparison procedure was performed. For comparisons, we used standardized results from the factor model.

In order to examine the relationship between children’s body-fat status and their eating behaviors, a linear regression using the backward method was calculated. The analysis was carried out separately for the groups of girls and boys. In the initial models, the dependent variable was body-fat percentage, while the predictors were eight dimensions of eating behaviors. At the end, only significant predictors were left in the models (see in the Results section).

To show the relationship between parents’ feeding styles and children’s eating behaviors, we calculated the Pearson correlation coefficient (bivariate correlation) between variables separately for four groups of children (sex × body-fat status).

## 3. Results

### 3.1. Children’s Nutritional Knowledge, Eating Habits and Behaviours, Parental Feeding Styles, and Body-Fat percentage (%BF) Descriptive Statistics

First, we present summary data for all variables measured both in children and parents (Table 1).

Analysis of SES (the family’s area of residence and parental level of education) and familial factors (the number of siblings) showed no significance for parental feeding practices and child food-related experiences.

### 3.2. Body-Fat Status of the Five-Year-Old Boys and Girls and Their Parents

We divided the children into two groups, girls and boys, based on body-fat percentage: healthy and overfat [39]. We also divided mothers and fathers into healthy and overfat groups according to the norms set for the measuring instrument used [40]. Descriptive statistics for the examined dependent variable in each group are shown in Table 2.

### 3.3. Determination of Body-Fat Status

**Figure 1:**
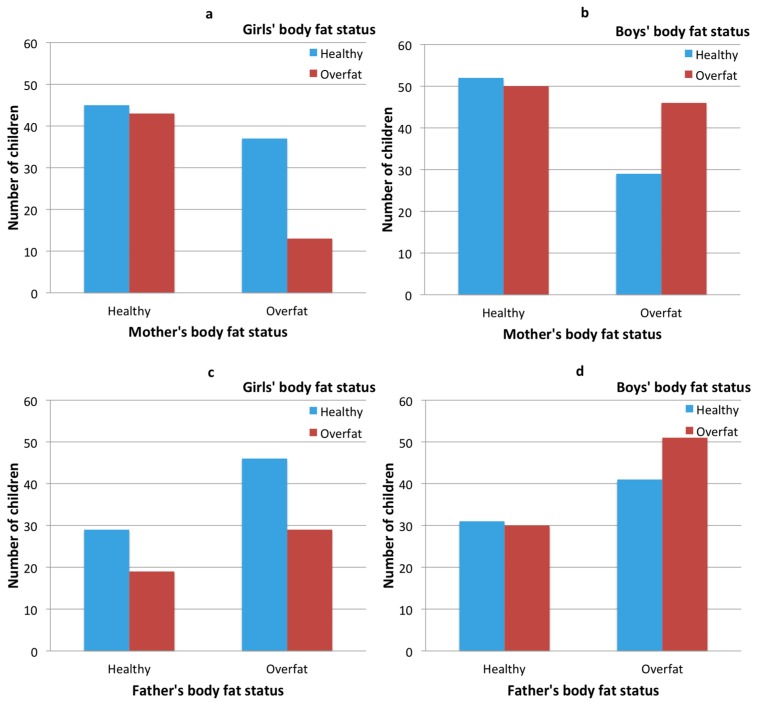
Clustered bar charts: children’s body fat and their parents’ body fat cross-tabulation—girls’ and their mothers’ body fat status (**a**), boys’ and their mothers’ body fat status (**b**), girls’ and their fathers’ body fat status (**c**) and boys’ and their fathers’ body fat status (**d**).

The results of the comparison of the numbers of healthy and overfat children (separately for girls and boys) in the healthy and overfat parents’ groups (separately for mothers and fathers; see Figure 1) indicate that significant differences only occur in the mother–daughter relationship (see Figure 1a). In the case of overweight mothers, overweight daughters are significantly less likely (chi-square = 6.91, *p* < 0.01). In other cases, the differences in frequencies in groups are not statistically significant.

### 3.4. Parents’ Body-Fat Status and their Feeding Styles

The results showed that significant differences between healthy and overfat mothers occur only for the encouragement feeding style: mothers with healthy body fat status (*M* = 4.21, SD = 0.57) used this style less often than did overfat mothers *(M* = 4.33, SD = 0.56; *t* = −1.88, *df* = 313, *p* < 0.05). In the case of fathers, differences appeared in the control over eating feeding style: fathers with healthy body fat status (*M* = 3.83, SD = 0.56) used this style more often than did overfat fathers *(M* = 3.67, SD = 0.63; *t* = 2.18, *df* = 274, *p* < 0.05). The results of the paired samples tests showed significant differences between mother’s instrumental feeding style (*M* = 2.31, SD = 0.83) and father’s instrumental feeding style (*M* = 2.43, SD = 0.88; *t* = −2.88, *df* = 355, *p* < 0.01); mother’s emotional feeding style (*M* = 2.09, SD = 0.86) and father’s emotional feeding style (*M* = 2.20, SD = 0.79; *t* = −2.72, *df* = 355, *p* < 0.01); and mother’s encouragement feeding style (*M* = 4.27, SD = 0.57) and father’s encouragement feeding style (*M* = 4.12, SD = 0.68; *t* = 4.26, *df* = 355, *p* < 0.01).

### 3.5. Children’s Body Fat Status and Their Nutrition Knowledge and Eating Habits

**Figure 2:**
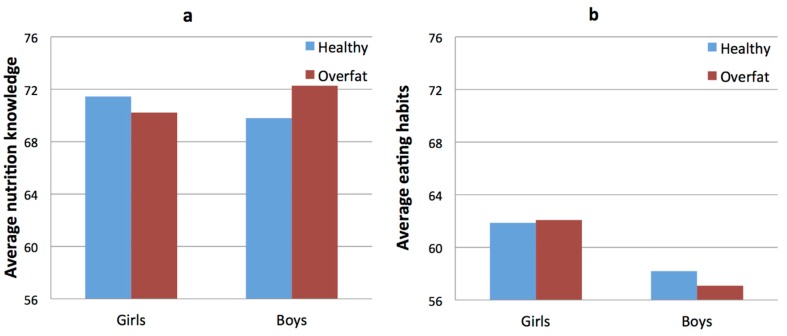
Average nutrition knowledge (**a**) and average eating habits (**b**) in groups of healthy and overfat girls and boys.

The results did not show significant main effects of sex or body fat status: there were no differences between boys and girls, neither in the healthy nor the overfat children, in average nutrition knowledge. However, an interaction effect between measured variables was demonstrated, *F*(1, 385) = 3.74, *p* = 0.05: The differences in average nutrition knowledge and eating habits depends on the sex of the children. Post hoc tests showed that overfat boys had a higher average knowledge of the nutritional value of foods (*M* = 72.27, SD = 7.62) than did healthy boys (*M* = 69.80, SD = 10.54; *t* = −1.98, *p* = 0.05)—this difference is not apparent in the group of girls. In the test of eating habits, the girls (*M* = 61.95, SD = 10.57) achieved higher scores compared to the boys (*M* = 57.61, SD = 11.26), *F*(1, 385) = 14.58, *p* < 0.01 (see Figure 2). However, there was no main effect for the body fat status variable or an interaction effect between the variables examined.

### 3.6. Children’s Body-Fat Status and Their Parents’ Feeding Styles

**Figure 3:**
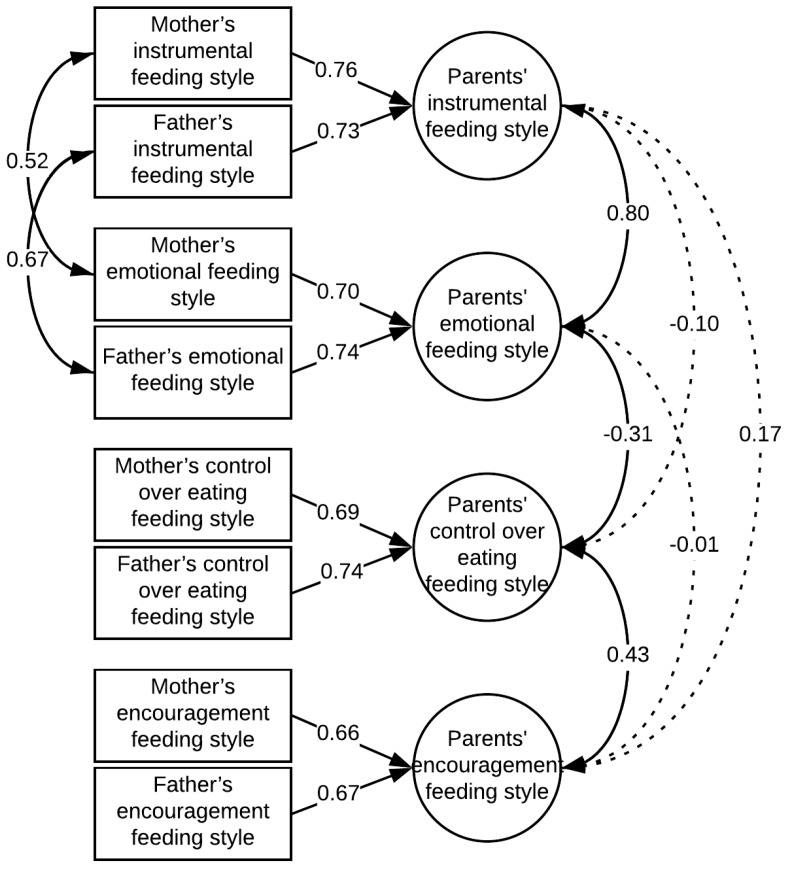
Parents’ feeding styles model. Standardized factor loadings and coefficients of covariance between variables are shown in the diagram.

Figure 3 shows the structural equation modeling results testing the measurement model of parents’ feeding style which assumed the compatibility of mothers’ and fathers’ tendencies in each of the four defined styles. The model proved to be well fitted to the data (*χ*^2^ = 23.35, *df* = 12, CFI = 0.99, TLI = 0.97, *RMSEA* = 0.052, SRMR = 0.024).

**Figure 4:**
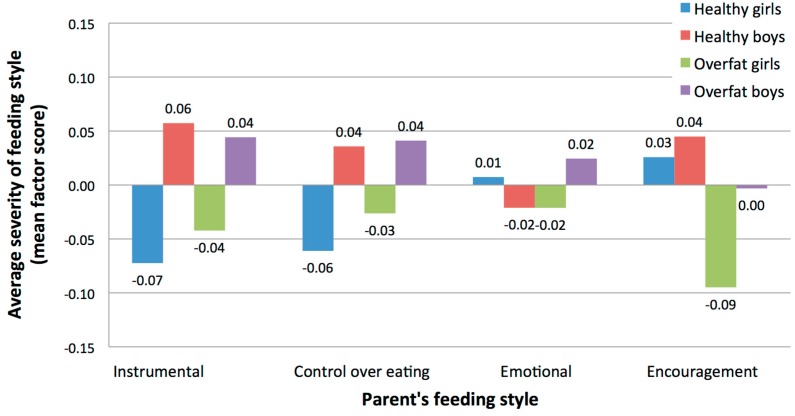
Average severity of parents’ feeding styles (standardized factor scores) in groups of healthy and overfat girls and boys.

The MANOVA results shows two significant main effects (significance of differences between groups in average severity of parents’ feeding styles): one for sex, the other for body-fat status (see Figure 4). For girls (*M* = −0.06, SD = 0.53), parents use the instrumental feeding style less often than for boys (*M* = 0.05, SD = 0.52), *F*(1, 356) = 3.61, *p* = 0.05. In the case of overweight children (*M* = −0.04, SD = 0.29), parents are less likely to use the encouragement style than for children without excess body-fat status (*M* = 0.04, SD = 0.31), *F*(1, 356) = 6.92, *p* = 0.01. However, post hoc tests showed that this difference is significant only in the group of girls.

### 3.7. Children’s Body-Fat Status and Their Eating Behaviors

The results of the linear regression using the backward method are shown in Table 3. In the group of girls, satiety responsiveness and food responsiveness explain 8% of the variance of body fat status [*Adjusted R*^2^ = 0.08, *F*(2, 160) = 8.08, *p* < 0.01], while in the group of boys, emotional undereating and emotional overeating explain 3% of the variance of body-fat status [*Adjusted R*^2^ = 0.03, *F*(2, 194) = 3.77, *p* < 0.05].

### 3.8. Parents’ Feeding Styles and Children’s Eating Behaviors

As shown in Table 4, instrumental and control over eating feeding styles positively correlate with food responsiveness and emotional overeating in the group of girls with healthy body-fat status and in the group of overfat boys. In the case of healthy boys, these styles only correlate with emotional overeating. In addition, enjoyment of food positively correlates with instrumental and control over eating–feeding styles only in the group of healthy girls; a desire to drink correlates with the control over eating–feeding style in the group of overfat girls and healthy boys; emotional undereating positively correlates with instrumental and control over eating–feeding styles in the overfat girls and boys group. The only statistically significant negative correlation occurred between the desire to drink and emotional feeding styles in the group of healthy boys.

## 4. Discussion

In this study, we examined nutritional knowledge, eating habits and appetite traits among Polish five-year-old boys and girls with and without excessive body fat status (healthy vs. overfat children) in the context of parental feeding styles and their parents’ body-fat status. We hypothesized that (a) the feeding styles preferred by parents determine children’s eating habits and shape their nutritional knowledge, consequently contributing to their body-fat status; and (b) given the gender and body-fat status of both the parents and children, distinct patterns of relationships are present between parental feeding styles and the eating habits and nutritional knowledge of girls and boys. The outcomes of the study partially confirm these hypothesis.

Our results revealed that overfat mothers, who used the encouragement feeding style more frequently, rarely had daughters with increased %BF. Parents of overfat girls, however, were less likely to apply the encouragement and instrumental feeding styles. These results were not confirmed among boys. Healthy and overfat girls presented healthier eating habits than boys, however, overfat boys had better nutritional knowledge. Children’s %BF was found to be linked with such eating behaviors as low satiety responsiveness and increased food responsiveness in girls as well as low emotional undereating and increased emotional overeating in boys. Feeding represents the earliest form of parental influence and it is often reported that sons and daughters are treated differently in this regard [42].

Two important phenomena have been observed in this research. Contrary to popular belief and previous studies [43,44], overfat women do not necessarily transmit unhealthy eating patterns to their children. Furthermore, parents are more preoccupied with managing the weight and eating habits of their daughters than of their sons, which is most probably linked to their awareness of the importance of social standards of physical attractiveness [45]. Both the %BF and gender of parents and children are important factors associated with parental feeding styles.

In line with the growing body of research indicating that increased maternal weight status does not inevitably determine childhood obesity [37,46], our research revealed that daughters of overfat mothers had a healthy %BF. Preoccupied that their daughters may develop a weight problem in the future, overfat mothers are possibly more cautious when feeding girls. Their concern may stem from their own challenging experiences related to elevated %BF: adverse health outcomes, struggles with dieting and controlling weight, and other personal issues. Moreover, as this result pertains only to girls, it is safe to assume that mothers’ preoccupation with their daughters’ weight emerges from their awareness of the great pressure that society places on women’s weight and the social value of thinness [30]. Being mindful of the importance of physical attractiveness, mothers are more careful in their food-related practices with girls.

Mothers’ investment in weight and eating issues, their perception of their child’s body mass index (BMI), and concern for their child’s weight are key factors predicting maternal feeding styles [29]. In our study, overfat mothers were more prone to encourage their children, regardless of their gender and %BF, to try a variety of foods. The encouragement feeding style is a pro-health approach and linked to healthy eating habits in children [47,48,49]. Early exposure to different types of nutrients and a variety of tastes and textures may lead to the broadening of the food preferences of a child who, therefore, will favor healthy meals. Aware of the various health and social disadvantages of obesity, overfat mothers possibly feel responsible for preemptively forming healthy eating habits as early as possible.

Interestingly, overfat fathers reported decreased control over their children’s food intake. Two possible explanations can be offered to explain this result. First, it is possible that overfat fathers may, in general, have lower control over their eating habits and, therefore, little control over their children’s food intake. Secondly, fathers may be less engaged in choosing and preparing meals for the family which, in Poland, is a task traditionally assigned to women [50]. Having control over a child’s eating is a feeding style positively associated with such nutritive eating patterns as frequent consumption of fruits, vegetables and breakfast as well as avoidance of high-energy-density food [47]. Unsurprisingly, lack of parental control over food intake has been linked to weight issues in children [37].

Additional to the aforementioned gender differences between mothers and fathers, feeding styles were determined by the child’s gender and %BF. Interestingly, parents of overfat daughters used the encouragement style less often. Based on the results of previous research projects, limited exposure to novel foods and lack of early experiences with a variety of flavours may contribute to the consolidation of unhealthy food preferences and, consequently, lead to an elevated risk of being overfat and/or obese. Children at an early age have a tendency to avoid novel foods [1] and they have an innate predisposition to prefer sweet and salty tastes [51], and energy-dense foods [52]. Cross-cultural research indicates that children, unsurprisingly, prefer fries, pizza and ice-cream over meals with better nutritional value [13,35,53] and that these preferences are fairly stable over time [13] unless modified by parents [54]. If not exposed to a variety of foods and encouraged to taste unfamiliar flavours, children’s preferences will remain limited and they will continue to favour food of low nutritional value like snacks and sugared drinks [55,56].

All parents of girls, regardless of their %BF, were less likely to use the instrumental feeding style. The instrumental feeding style is a non-nutritive behavior used by parents to regulate a child’s behavior (e.g., rewarding the child with food) which may create the risk of the child developing the habit of eating despite the absence of hunger [57] and a preference for high energy-density food [47]. The instrumental feeding style has been associated with increased food responsiveness and eating snacks by children [48,58] and food responsiveness has been positively linked to children’s weight status [59]. A parental preoccupation with maintaining socially-desirable thinness in girls is possibly reflected in avoiding feeding styles that may promote non-nutritive eating habits and, thus, minimizing the risk of being overfat or obese.

In our study, the risk of increased %BF was associated with eating behaviours and appetite regulation. In line with previous evidence, which indicated a positive association between weight and food approach-related appetite traits and a negative association with food avoidance-related appetite traits [59,60,61], excess food responsiveness and low satiety responsiveness predicted elevated %BF in girls, while increased emotional overeating and low emotional undereating predicted elevated %BF in boys. Girls with high %BF had higher appetites, enjoyed external eating, and over-valued food, whereas boys with increased %BF showed a stronger preference for eating during emotional stress.

Gender differences also emerged in behaviourally observed eating habits. Girls, regardless of their %BF, showed healthier eating habits than boys. Previous studies confirm that girls like vegetables and fruits more than do their male peers, who were found to rather prefer fatty food, sugar, processed products and meat [35,62,63,64]. Cross-cultural studies [65] indicated that women focus on healthy eating choices (avoiding fat and salt as well as consuming fruits and fibre) and greater weight control as they are influenced by social expectations regarding dieting and an attractive, slim physical appearance. Our results suggest that even five-year-old girls are probably aware of the social importance of diet and, thus, internalize the eating habits socially expected of women. For men, healthy eating is often of less importance and, thus, they follow healthy eating recommendations less often [65]. Furthermore, healthy eating or particular types of food can be associated with typically feminine behaviour, so men who want to follow “male” stereotypes may avoid health behaviours often attributed to women [31,32]. This could explain the more ambivalent attitude of men towards healthy dietary behaviours [33]. Interestingly, the transmission of gender role stereotypes has been found to be the strongest for the father–son relationship [66]. Furthermore, boys may prefer energy-dense foods as their energy requirements are greater than those of girls [62].

Interestingly, overfat boys had greater nutritional knowledge than healthy boys and all girls. It is possible that their expanded knowledge results from parental effort to prevent sons from gaining further weight by increasing their awareness of the nutritional value of meals. Children as young as six understand the relationship between eating and weight [67], although research indicates that obese and non-obese children have similar nutritional knowledge [68] and that nutritional knowledge has a small impact on eating behaviours [69,70]. Therefore, while planning prevention interventions for children at risk of obesity, expanding nutritional knowledge should be predominantly supported by strategies modelling healthy eating patterns [71].

One of the main strengths of this research concerns the use of %BF instead of BMI. Although the use of BMI is very common, its diagnostic value for adiposity has been demonstrated to be unreliable and insufficient [72]. BMI is not an accurate measure of body fat percentage (%BF), and thus can lead to serious miscalculations [73]—excessive %BF can be present in persons with BMI within normal limits [74]. Furthermore, excess %BF is a more accurate predictor of health risks than BMI [72]. Substantial discrepancies in the assessment of body-fat status based on BMI have led to a lot of difficulties in determining expected body mass ranges or preparing dietary or training recommendations [74]. These difficulties are particularly evident in children, whose weight/height ratios change very rapidly. Thus, we have used %BF as the most reliable indicator of body fat distribution [38]. An additional advantage of our research concerns the fact that both parents were assessed, which allowed us to compare and contrast the parental feeding styles of mothers and fathers. A majority of studies has rarely involved both parents, and usually mothers are the only participants. The results of our study show that the gender of both the parents and the child are important for understanding the trans-generational transmission of eating habits and preferences.

## 5. Limitations

Unfortunately, one of the greatest limitations of this study concerns its inability to assess the degree to which the nutritional knowledge of overfat boys and the healthier eating habits demonstrated by girls are manifested during real meals. Primarily, we used self-report assessments of parental feeding styles, a parent-completed questionnaire regarding children’s eating behaviours, and a game-interview with the child to assess eating habits and nutritional knowledge. Although these methods are widely used in research, they do not provide complete information on parents’ and children’s behaviours in typical eating situations. In future research, direct observations should be included. It is important to bear in mind that the children who participated in our study were fed by their parents and/or caregivers, as they were only five years old. Thus, they had very little control over the quality of the meals served. Therefore, any naturalistic observation of their food preferences and eating habits would be limited.

Furthermore, as children and parents were assessed only at one point in time, this research failed to provide evidence of causal effects and to capture the dynamics between the investigated factors. For instance, we theorized that a decrease in encouragement to eat among parents of overfat daughters could be the cause of their increased %BF. Nonetheless, longitudinal studies are required to confirm this assumption. Therefore, the participants of this study will be assessed again when they turn eight years old. Children at the age of eight years attend elementary school where their parents have limited control over the quality and quantity of food consumed at school (e.g., food bought in the school’s cafeteria), and thus their food preferences and choices are more apparent.

## 6. Conclusions

In order to contribute to the growing body of research on the trans-generational transmission of obesity risk, we focused on investigating the significance of parent–child dietary intake patterns on the development of children’s eating habits, nutritional knowledge, eating behaviors and %BF. In doing so, important phenomena have been observed. Overfat women do not necessarily transmit unhealthy eating patterns to their children. Health afflictions, struggles with dieting and controlling weight, and an awareness of the significance of social female beauty standards may incline overfat mothers to be cautious about the quality and quantity of meals provided to their daughters and to use feeding styles which contribute to the development of healthy eating patterns. Furthermore, the %BF and gender of parents and children are important factors associated with this process. Parents’ greater emphasis on managing the weight and eating habits of daughters (rather than sons) was apparent in their choice of pro-health feeding styles. The importance of thinness reflected itself not only in parental caution in feeding-related practices but also in a preference for healthy eating habits among the five-year-old girls examined. Therefore, we conclude that social standards of female physical attractiveness are transmitted early in trans-generational processes within the context of parent–daughter dietary intake interactions.

## Figures and Tables

**Table 1:** Descriptive statistics for the variables measured in the study (*n*—number of observations = 387).

Variable	Score Range	Min.	Max.	*M*	SD
Child’s % body fat	–	13.10	51.60	22.11	4.94
Mother’s % body fat	–	8.40	61.40	31.46	8.94
Father’s % body fat	–	4.20	61.70	22.59	8.36
Mother’s instrumental feeding style	1–6	1.00	5.50	2.32	0.83
Mother’s control over eating feeding style	1–6	2.10	5.40	3.82	0.56
Mother’s emotional feeding style	1–6	1.00	5.60	2.11	0.86
Mother’s encouragement feeding style	1–6	1.38	6.00	4.26	0.57
Father’s instrumental feeding style	1–6	1.00	5.50	2.43	0.88
Father’s control over eating feeding style	1–6	1.50	6.00	3.72	0.59
Father’s emotional feeding style	1–6	1.00	5.00	2.20	0.79
Father’s encouragement feeding style	1–6	1.00	5.63	4.12	0.68
Child’s nutrition knowledge	0–90	36.67	85.17	71.03	9.27
Child’s eating habits	0–90	27.00	90.00	59.58	11.15
Food responsiveness	5–30	5.00	30.00	11.65	3.89
Emotional overeating	4–24	4.00	20.00	9.23	2.59
Enjoyment of food	4–24	4.00	24.00	14.03	3.44
Desire to drink	3–18	3.00	18.00	9.94	3.25
Satiety responsiveness	5–30	6.00	27.00	17.24	3.32
Slowness in eating	4–24	4.00	24.00	13.92	3.55
Emotional undereating	4–24	4.00	23.00	12.58	3.22
Food fussiness	6–36	8.00	36.00	22.36	5.82

Notes: Min.—minimum value; Max.—maximum value; *M*—mean; SD—standard deviation.

**Table 2:** Descriptive statistics for body-fat percentage for healthy and overfat girls and boys and for healthy and overfat mothers and fathers of children being studied.

Participants	Healthy					Overfat				
	*n*	Min.	Max.	*M*	SD	*n*	Min.	Max.	*M*	SD
Girls	101	16.10	22.90	20.96	1.40	73	23.00	51.60	27.55	5.33
Boys	100	13.10	19.90	17.29	1.84	113	20.00	39.90	23.90	4.07
Mothers	190	8.40	32.40	25.50	5.20	125	33.00	61.60	40.27	5.60
Fathers	109	4.20	21.90	14.75	4.35	167	20.20	61.70	27.72	6.02

Notes: *n*—number of observations; Min.—minimum value; Max.—maximum value; *M*—mean; SD—standard deviation.

**Table 3:** Result of regression for the relationship between selected eating behaviors and body fat percentage in the groups of girls and boys.

Predictor	Unstandardized Coefficients		Standardized Coefficients (Beta)	*t*	*p*-Value
	*B*	Standard Error			
**Girls**					
Food responsiveness	0.29	0.09	0.26	3.34	0.01
Satiety responsiveness	−0.18	0.11	−0.13	−1.66	0.10
**Boys**					
Emotional overeating	0.32	0.14	0.17	2.34	0.02
Emotional undereating	−0.23	0.11	−0.15	−2.06	0.04

Note: Dependent variable: child’s % body fat.

**Table 4:** Correlations between parents’ feeding styles and children’s eating behaviors in groups of boys and girls with healthy or overfat body-fat status.

Child’s Sex	Child’s Body Fat Status	Parents’ Feeding Style	FR	EO	EF	DD	SR	SE	EU	FF
Girls	Healthy (*n* = 96)	Instrumental	0.46 **	0.45 **	0.22 *	0.14	0.01	−0.03	0.08	0.03
		Control over eating	0.47 **	0.46 **	0.27 **	0.04	−0.12	−0.05	0.04	−0.14
		Emotional	−0.06	−0.09	−0.11	−0.03	0.09	0.01	−0.04	0.17
		Encouragement	0.09	0.03	0.12	0.17	0.16	0.08	0.01	0.02
	Overfat (*n* = 65)	Instrumental	0.10	0.13	−0.07	0.17	0.16	0.09	0.33 **	0.21
		Control over eating	0.19	0.21	−0.04	0.34 **	0.15	0.03	0.31 *	0.16
		Emotional	−0.03	−0.06	0.17	−0.22	−0.12	−0.02	0.08	0.04
		Encouragement	−0.06	−0.03	0.03	0.01	0.01	0.13	0.27 *	−0.07
Boys	Healthy (*n* = 90)	Instrumental	0.11	0.33 **	−0.06	0.18	0.08	0.08	0.16	0.25 *
		Control over eating	0.17	0.32 **	−0.02	0.34 **	0.07	0.09	0.10	0.28 **
		Emotional	0.02	−0.12	−0.09	−0.31 **	0.09	0.07	0.09	0.01
		Encouragement	−0.15	−0.10	−0.08	−0.15	0.01	0.14	0.07	0.06
	Overfat (*n* = 105)	Instrumental	0.24 *	0.44 **	0.03	0.17	0.08	0.18	0.35 **	0.01
		Control over eating	0.25 **	0.46 **	−0.01	0.16	0.02	0.13	0.28 **	−0.05
		Emotional	−0.04	−0.25 *	0.01	−0.02	−0.02	0.02	−0.19	0.06
		Encouragement	−0.08	−0.11	0.15	0.10	0.03	0.07	0.20 *	−0.09

Notes: * *p* < 0.05, ** *p* < 0.01. Abbreviations: FR—food responsiveness; EO—emotional overeating; EF—enjoyment of food; DD—desire to drink; SR—satiety responsiveness; SE—slowness in eating; EU—emotional undereating; FF—food fussiness.
